# Quantifying Isoniazid Levels in Small Hair Samples: A Novel Method for Assessing Adherence during the Treatment of Latent and Active Tuberculosis

**DOI:** 10.1371/journal.pone.0155887

**Published:** 2016-05-18

**Authors:** Roy Gerona, Anita Wen, Aaron T. Chin, Catherine A. Koss, Peter Bacchetti, John Metcalfe, Monica Gandhi

**Affiliations:** 1 Department of Obstetrics, Gynecology and Reproductive Sciences, University of California San Francisco (UCSF), San Francisco, CA, United States of America; 2 Department of Medicine, Division of Pulmonary and Critical Care Medicine, University of California San Francisco (UCSF), San Francisco, CA, United States of America; 3 Department of Medicine, Division of HIV, Infectious Diseases, and Global Medicine, University of California San Francisco (UCSF), San Francisco, CA, United States of America; 4 Department of Epidemiology and Biostatistics, University of California San Francisco (UCSF), San Francisco, CA, United States of America; University of Cape Town, SOUTH AFRICA

## Abstract

**Background:**

Tuberculosis (TB) is the leading cause of death from an infectious pathogen worldwide and the most prevalent opportunistic infection in people living with HIV. Isoniazid preventive therapy (IPT) reduces the incidence of active TB and reduces morbidity and mortality in HIV-infected patients independently of antiretroviral therapy. However, treatment of latent or active TB is lengthy and inter-patient variability in pharmacokinetics and adherence common. Current methods of assessing adherence to TB treatment using drug levels in plasma or urine assess short-term exposure and pose logistical challenges. Drug concentrations in hair assess long-term exposure and have demonstrated pharmacodynamic relevance in HIV.

**Methods:**

A large hair sample from a patient with active TB was obtained for assay development. Methods to pulverize hair and extract isoniazid were optimized and then the drug detected by liquid chromatography/ tandem mass spectrometry (LC/MS-MS). The method was validated for specificity, accuracy, precision, recovery, linearity and stability to establish the assay’s suitability for therapeutic drug monitoring (TDM). Hair samples from patients on directly-observe isoniazid-based latent or active TB therapy from the San Francisco Department of Public Health TB clinic were then tested.

**Results:**

Our LC/MS-MS-based assay detected isoniazid in quantities as low as 0.02ng/mg using 10–25 strands hair. Concentrations in spiked samples demonstrated linearity from 0.05–50ng/mg. Assay precision and accuracy for spiked quality-control samples were high, with an overall recovery rate of 79.5%. In 18 patients with latent or active TB on treatment, isoniazid was detected across a wide linear dynamic range.

**Conclusions:**

An LC-MS/MS-based assay to quantify isoniazid levels in hair with performance characteristics suitable for TDM was developed and validated. Hair concentrations of isoniazid assess long-term exposure and may be useful for monitoring adherence to latent or active TB treatment in the setting of HIV.

## Introduction

Tuberculosis (TB) remains one of the greatest threats to public health today, surpassing HIV as the leading cause of death from an infectious pathogen in 2015 [1]. TB is also the leading cause of death among HIV-infected individuals worldwide and the provision of isoniazid preventive therapy (IPT) to those with HIV reduces the risk of HIV-associated illness or death significantly and independently of antiretroviral therapy (ART) [2, 3]. Yet, controlling the TB epidemic has been threatened by high rates of treatment default and failure. The requirement of prolonged treatment courses for latent and active TB threatens sustained adherence, and considerable inter-patient pharmacokinetic (PK) variability for commonly-used TB medications, including isoniazid (INH), may contribute to the emergence of resistance [4, 5] and adverse treatment outcomes.

Given the importance of monitoring adherence and exposure to anti-TB medications during the treatment of both latent TB infection (LTBI) and active disease, the routine incorporation of therapeutic drug monitoring (TDM), where drug levels are monitored in a biomatrix, has been recommended[6]. Pharmacologic exposure measures are important in disease states such as TB, where no accurate individual-level surrogate biomarkers for assessing early preventative or curative responses exist. Ensuring optimal TB medication adherence/exposure is even more critical in HIV-infected patients, in whom the risk of low drug exposure, drug-drug interactions, adherence difficulties, and acquired drug resistance is highest [7–10]. TDM using single or multiple plasma levels indicates only recent exposure and requires phlebotomy, a cold chain for storage and shipment, and patient accuracy in reporting the time of the last dose consumed. Monitoring urine concentrations of anti-TB medications holds promise, but also assesses drug or metabolite exposure over short periods of time and requires a cold chain. TDM via drug levels in dried blood spot (DBS) avoids logistical issues associated with plasma assays [11], although instability of INH in DBS, variability in the sample method, and lack of assay standardization can introduce error [12]. A low-cost, noninvasive, easily-collected, alternative method for assessing adherence and exposure to TB drugs among HIV-infected and uninfected patients could provide an important clinical tool for both LTBI and active TB treatment.

Levels of medications in hair reflect drug uptake from the systemic circulation over weeks to months [13] and surpass single plasma assays or DBS monitoring in reflecting long-term exposure. Hair collection is noninvasive and does not require specific skills, sterile equipment, specialized storage conditions, a cold chain, or biohazardous precautions. Our group has cultivated expertise in monitoring antiretroviral (ARV) exposure during HIV treatment and prevention via assaying ARV concentrations in small-volume hair samples [14–26]. We have found that hair levels of ARVs correlate strongly with areas-under-the-plasma concentration-time-curves (AUCs) from intensive PK studies [20], are the strongest independent predictor of treatment outcomes in HIV disease [15–18, 23, 24], and display linear relationships with dose [21]. Monitoring anti-TB drugs in hair could provide a long-term measure of exposure with feasibility advantages in the resource-poor setting. We describe here the development and validation of an assay to measure isoniazid in small hair samples using liquid chromatography- tandem mass spectrometry (LC-MS/MS).

## Materials and Methods

### Shaved head sample for assay development

In order to develop and optimize laboratory assays for measuring anti-TB drugs in hair, we require large volumes of hair and therefore shave the heads of patients on LTBI or active TB treatment. We recruited one HIV-infected patient from San Francisco General Hospital with active pulmonary TB who was on INH, rifampin, pyrazinamide, and ethambutol for initial assay development. Of note, this patient was also on dolutegravir (dosed twice daily), tenofovir disoproxil fumarate (TDF) and emtricitabine (FTC) for the simultaneous treatment of his HIV infection. The protocol and written consent form for shaving the heads of individuals on anti-TB therapy were approved by the UCSF Committee on Human Research (UCSF CHR #14–14609) prior to recruitment. Upon obtaining written informed consent, we refer patients to a barber employed by the study who cuts each person’s scalp hair down to a total length of approximately 1 inch (~2.5 centimeters (cm)) and then completely shaves off the remaining hair for the assay optimization work.

### Cut hair samples for assay validation

We then recruited 18 patients who were on INH either for LTBI (n = 7) or for active TB treatment (n = 11) from the San Francisco Department of Public Health TB Clinic for the collection of 200–300 strands of hair (~20–30 mg) for assay validation. Of the 11 patients on active TB therapy, all were on directly observed therapy (DOT). Of the 7 patients treated for LTBI, 2 were on daily INH (300mg) and not on DOT; five were on intermittent once weekly LTBI treatment (INH 900mg and rifapentine once weekly) and were all on DOT. All patients provided written informed consent and the protocol was approved by the UCSF Committee on Human Research (UCSF CHR #14–14609) prior to the collection of smaller hair samples.

Hair sample collection has been previously described [22]. Briefly, a small thatch of hair is isolated from the occipital region of the scalp and cut as close to the scalp as possible. The cut thatch of hair is placed inside a piece of tin foil with a patient study ID label taping the distal end of the hair thatch (furthest away from the scalp) to the foil to mark directionality. The hair sample is then stored in a plastic bag at room temperature and in a dark place prior to analysis. Of note, we collected 200–300 strands of hair for assay validation in this study, but required only 10% of that (10–25 strands) for analysis; the remainder of each hair sample was stored for repeat runs and future experiments.

Table 1 describes the characteristics of the one individual in the shaved head study (patient SH-001) and the 18 individuals with active or latent TB from whom small samples of hair were cut. Hair collection from these individuals was conducted from July-September 2015.

**Table 1:** Characteristics of patients on INH recruited July-September 2015 for INH measurements in small hair samples.

Sample ID	Age	Gender	Ethnicity	Active, Latent or Active TB	DOT?	HIV Positive?	INH Duration (months)	INH Dosage (mg)	Concomitant Anti-TB drugs
**SH-001**	51	M	African American	Active	Yes	Y	8	300mg	Rifampin,
**TB-003**	41	F	Hispanic	Active	Yes	N	2	300	Rifampin, Ethambutol
**TB-004**	40	M	Asian/Filipino	Latent	Yes	N	2	900 per week	Rifapentine once weekly
**TB-005**	56	M	Asian/Filipino	Active	Yes	N	7.5	300	Rifampin, Ethambutol
**TB-006**	53	M	Asian	Active	Yes	N	6.5	300	Rifampin, Ethambutol, Pyrazinamide
**TB-007**	3	M	Asian	Active	Yes	N	4	200	Rifampin, Ethambutol, Pyrazinamide
**TB-008**	66	M	Caucasian	Active	Yes	N	2.5	300	Rifampin, Ethambutol, Pyrazinamide
**TB-009**	47	M	Caucasian	Active	Yes	Y	4.5	300	Rifampin, Ethambutol, Pyrazinamide
**TB-010**	34	F	Asian/Chinese	Active	Yes	N	3.5	300	Rifampin, Ethambutol, Pyrazinamide
**TB-011**	76	F	Asian	Active	Yes	N	9	300	Rifampin, Ethambutol, Pyrazinamide
**TB-012**	55	M	Asian	Active	Yes	N	4	300	Rifampin, Ethambutol, Pyrazinamide
**TB-013**	46	F	Asian/Mongolian	Latent	No	N	2	300	Rifampin
**TB-014**	66	M	Asian/Chinese	Active	Yes	N	4	300	Pyrazinamide, Ethambutol
**TB-015**	18	M	Hispanic	Latent	No	Never tested	4.5	300	None
**TB-016**	75	M	Asian	Latent	Yes	N	1	900 per week	Rifapentine once weekly
**TB-017**	26	M	Asian	Active	Yes	N	1.5	300	Rifampin, Ethambutol, Pyrazinamide
**TB-018**	54	M	Hispanic	Latent	Yes	N	1	900 per week	Rifapentine once weekly
**TB-020**	25	M	Hispanic	Latent	Yes	N	2	900 per week	Rifapentine once weekly
**TB-021**	15	F	Filipina	Latent	Yes	N	2.5	900 per week	Rifapentine once weekly

### Methods to analyze INH in hair

#### Chemicals

We purchased reference standards for isoniazid (98% chemical purity) and isoniazid-d4 (internal standard, 98% chemical purity, 98.4% isotopic purity) from Toronto Research Chemicals (Ontario, Canada). We obtained analytical grade water, methanol, and acetonitrile from Honeywell Burdick and Jackson (Muskegon, MI). We prepared stock solutions of the standard and internal standard at 1 milligram (mg)/milliliter (mL), aliquoted to 1mL portions in amber vials and stored at -80°C. All calibration standards, ranging in concentration from 0.05 to 50 nanograms (ng)/mg hair, were prepared from the stock solution.

#### Isoniazid Analysis

We used an Agilent LC 1260 (Sta. Cruz, CA) equipped with a binary pump and an AB Sciex API 5500 (Foster City, CA) as the analytical platform for our method. We pulverized human hair samples (2 mg, about 10–25 one centimeter strands) using an Omni Bead Ruptor^®^ homogenizer (Kennesaw, GA) and extracted the pulverized hair samples in methanol. The extracts (10 microliter (uL)) were then separated on a Phenomenex Synergi Polar-RP (2.1 x 100 mm, 2.5 μm particle size) column (Torrance, CA) using water with 0.2% (v/v) formic acid as mobile phase at a flow rate of 0.4 mL/min. We monitored INH at multiple reaction mode using an electrospray ion source operated in positive polarity. We used two transitions to monitor INH: 138.1 > 79.0 m/z (quantifier) and 138.1 >120.9 m/z (qualifier).

We analyzed the data obtained from our LC-MS/MS runs using AB Sciex Analyst 1.6 and AB Sciex MultiQuant 2.1 software packages (Foster City, CA). We identified and confirmed INH based on its retention time and the peak area ratio between its two transitions. We quantified INH in hair samples by isotope dilution method using INH-d4 as internal standard. We monitored INH-d4 using the transition 142.1 > 82.9 m/z.

### Method validation

To validate our method, we assessed its performance in terms of its specificity, precision, accuracy, linearity, recovery and stability. To assess specificity, we analyzed nine different lots of hair samples from subjects not on INH to determine if there are endogenous components of hair that could interfere with INH detection. We conducted precision, accuracy, and recovery studies by running three different concentrations of INH (low = 0.2 ng/mg, medium = 2.0 ng/mg, high = 20.0 ng/mg) spiked into a matrix blank. We verified that hair derived from subjects not on INH exhibited no INH signal as matrix blank. For within run precision, we ran five samples of each concentration within a batch while three batches of five samples of each concentration were run over three separate days to calculate between-run precision. Accuracy and recovery for each sample run were calculated along with precision.

We performed linearity studies by running the calibration standards (0.05–50 ng/mg) five separate times over separate days and assessing the linear regression coefficient of the calibration plot obtained in each run. We assessed the stability of the extracts obtained from blank hair samples spiked with different levels of INH (low, medium, high) during incubation at three different temperatures (25°C, 4°C and -80°C) for 21 days. We checked the level of INH in each extract at days 1, 2, 3, 7, 10, 14, 17 and 21 or until at least one of the extracts at a certain incubation temperature was <80% its initial value.

### INH measurement in patient samples

We used the above-described assay to measure INH in hair samples from 18 recruited patients on INH. Duplicate samples of each patient were also run.

## Results

### INH detection in hair

**Fig 1:**
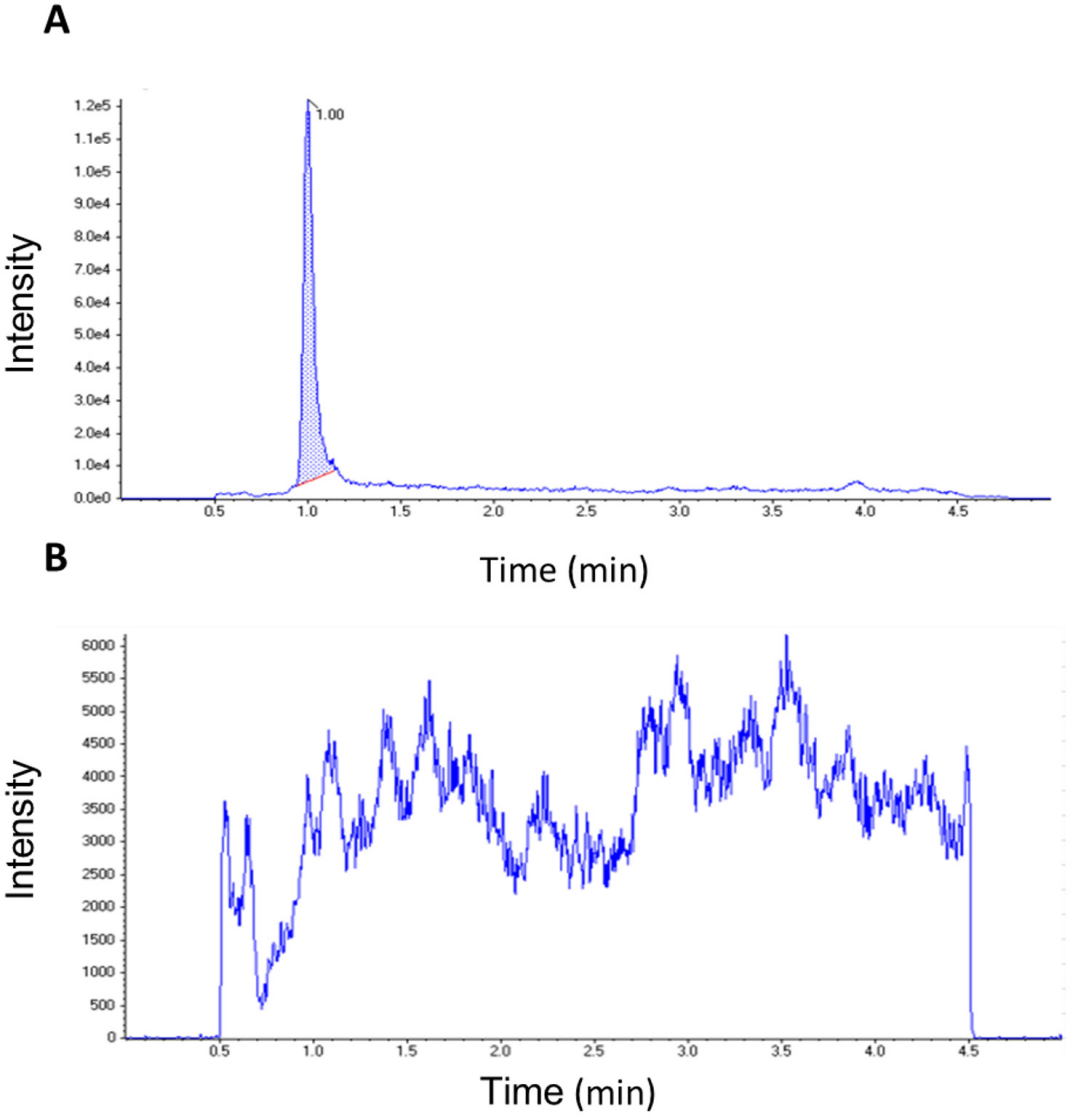
Representative extracted ion chromatogram of INH in small hair samples from a patient on INH (A) and a patient not taking INH (B).

We successfully developed a rapid and sensitive LC-MS/MS method for detecting and quantifying INH in small hair samples (~2 mg, or 10–25 strands of hair one cm in length). Fig 1 shows the INH peak in a typical LC/MS/MS chromatogram when the drug was extracted from a patient hair sample (patient TB-007). INH consistently elutes at 1.00 ± 0.05 min in a five-minute isocratic reversed phase chromatographic separation. INH in hair could be detected as low as 0.02 ng/mg (limit of detection, LOD) using 2 mg of hair, which is sensitive enough to detect INH in a range of patient hair samples.

### Method validation

No significant endogenous interfering peaks for isoniazid (i.e. peaks with signal to noise ratio ≥ 3) were observed in the chromatograms of the blank human hair samples (Fig 1). The spiked INH hair samples were linear from 0.05 ng/mg (lower limit of quantification, LLOQ) to 50 ng/mg (upper limit of quantification, ULOQ). The average linear regression coefficient we established is 0.998 from five separate linearity measurements. The assay precision (% coefficient of variation (CV)) and accuracy (relative error (RE)%) for all three levels of spiked hair samples were <15% (Table 2) while the recovery ranges between 71% and 84% with an over-all recovery rate of 79.5%. The recovery rates for all three levels of spiked hair samples are reproducible with precision (%CV) <10% (Table 2). Extracts obtained from spiked hair samples were stable for 1, 3, and 10 days at 25°C, 4°C and -80°C, respectively.

**Table 2:** Validation of method accuracy, precision and recovery for the analysis of isoniazid in small hair samples.

Concentration (ng/mg)	Precision (%CV)		Accuracy (%RE)		Recovery	
	Intraday	Interday	Intraday	Interday	Recovery (%)	%CV
**0.2**	9.21	14.75	-4.33	-3.27	84.05	5.55
**2.0**	5.03	12.32	-4.25	2.27	71.18	7.80
**20.0**	2.40	7.47	-6.69	-7.20	83.22	8.41

### INH detection in patient hair samples

**Fig 2:**
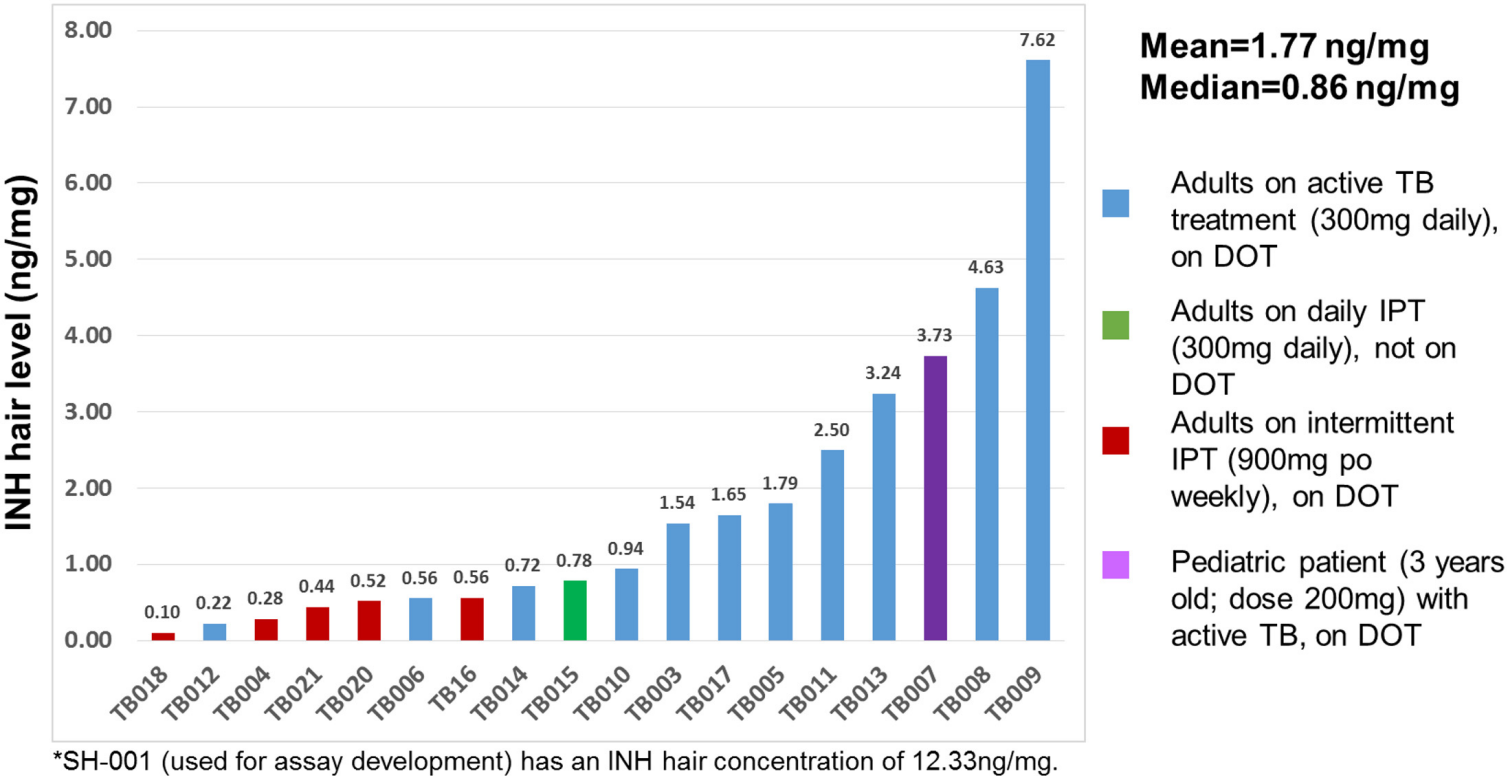
INH concentrations in 18 hair samples from patients recruited from the San Francisco Department of Public Health TB Clinic (11 with active TB; 7 with latent TB).

We detected INH in all patients recruited for our clinical validation study. Fig 2 shows the range of INH concentrations in 18 small hair samples for patients on either LTBI or active TB treatment. The mean, geometric mean, and median of INH hair concentrations were 1.77, 1.01, 0.86 ng/mg, respectively.

## Discussion

In this study, we describe the development of a novel LC-MS/MS based method to analyze INH in hair which is sensitive (LLOQ 0.05 ng/mg), precise (%CV <15%), reproducible, and accurate (RE%<10%). In an accompanying clinical validation study, we were able to isolate, identify, and quantify INH from small hair samples (~10–25 strands each) from patients on active or latent TB treatment over a wide linear dynamic range.

To our knowledge, this is the first report on the development and initial validation of a method for the direct analysis of INH in hair. A previous study reported the analysis of INH and acetyl-INH in hair samples from 40 patients[27]. However, the study did not report on the validation of the method and measured INH indirectly as its formyl-INH derivative. Direct measurement of analytes in biological matrices facilitates faster turn-around time with higher recovery rates; derivatization adds a step in the assay (prolonging it) where further loss of the analyte can be incurred. The assay we developed and validated only requires 60 minutes from hair pulverization to completion of the LC-MS/MS run. Efficient pulverization of the hair samples via the Omni Bead disruptor^®^ facilitated the direct extraction of INH via methanol with minimal incubation time. Methanol also facilitated the precipitation of proteins from pulverized hair. This simple method of extraction is consistent with the most common method of extracting INH from plasma samples [28–31], which also uses methanol as the agent of protein precipitation.

INH is a highly polar and basic compound. In reversed-phase (C18, C8) columns it elutes very close to the void volume even with a highly aqueous mobile phase [29], which may cause assay interference as other unretained matrix components may co-elute. We were able to improve the retention of INH using a polar RP column after testing several reversed-phase columns (Phenomenex Kinetex C18, Agilent ZORBAX Extend- C18, Agilent Poroshell 120). The polar group end cap of the polar RP column facilitated an interaction with INH that allowed for better retention. A narrow symmetrical peak was consistently obtained for INH within a narrow range of retention times (0.95–1.05 minute, Fig 1). The method is highly selective for INH as no peaks were detected in nine different lots of blank hair human samples (Fig 1). Likewise, within-run (intraday) and between-run (interday) precision and accuracy established for the method were all within the acceptance criteria set by the Division of AIDS-Clinical Pharmacology and Quality Assurance Program [32] based on the Food and Drug Administration (FDA) guidelines[33].

The standard curve established for the detection of INH in hair in this study has a wider linear dynamic range (0.05–50 ng/mg vs 0.05–1 ng/mg) than the range previously reported for formyl-INH and acetyl-INH [27]. The LLOQ established for the assay (0.05 ng/mg) is 2 times lower than the lowest measured INH level in the patient hair samples (Fig 1) and five times higher than the assay’s LOD. A wide variation in the concentration of INH in hair was observed in the 18 patients on active and latent TB treatment in this study. However, all measured INH levels fell within the linear calibration range. These performance characteristics—and the ability of the assay to distinguish inter-individual variability in drug levels (which is expected in the context of real-world adherence- establish the assay’s suitability for TDM.

Although only five patients in our study were receiving a lower cumulative dose of INH (900mg weekly), our studies suggest a dose-concentration relationship (Fig 2). The wide variation in INH levels may partially be attributable to pharmacogenetic differences among patients in their arylamine N-acetyltrasferase (NAT-2) genotype, a polymorphic gene that encodes for the primary enzyme that metabolizes INH [34]. A previous study that investigated the areas under the concentration-time curve for plasma INH 12 hours after dosing found a highly significant association with the NAT-2 genotype [35]. Future studies by our group will investigate the relationship between NAT-2 genotype and hair INH levels and the effects of different washing conditions and temperature exposure on the INH concentrations in hair. Finally, a more comprehensive investigation of these measures as a marker of adherence/exposure to latent or active TB treatment in large clinical cohorts of HIV-infected individuals is underway.

Given that INH is a critical, first-line component of both LTBI and active TB regimens, the ability to monitor this molecule in small hair samples may have potential utility a non-invasive measure of adherence and exposure. Moreover, the ability to extract INH from only 10–25 strands of hair should enhance acceptability of hair collection. Additional feasibility advantages to hair monitoring in resource-limited settings (RLS), where rates of TB infection are highest, include ease of hair collection, transport, and storage. Indeed, we have seen high rates of feasibility and acceptability of hair collection using small samples of hair from patients with HIV infection in multiple settings [22–24].

Since pharmacologic measures reflect both adherence and pharmacokinetics, concentrations of TB drugs in hair may provide an integrated measure of behavior and biology. Given the substantial problem with maintaining adherence to prolonged courses of TB treatment, despite implementation of the WHO Directly Observed Therapy-Short course strategy, in combination with considerable inter-individual pharmacokinetic variability of commonly-used TB medications [4, 5], a novel method of TDM that reflects both adherence and PK should be helpful to the field. In conclusion, we describe here the development and initial validation of a novel assay to analyze INH concentrations in small hair samples. Additional studies to evaluate the predictive utility of hair concentrations of INH on outcomes in HIV-infected patients on LTBI therapy and active TB treatment are ongoing.
